# 74325 Vast sex-specific differences in transcriptional landscapes of pancreatic neuroendocrine tumors

**DOI:** 10.1017/cts.2021.664

**Published:** 2021-03-30

**Authors:** Nikolay A. Ivanov, Thomas J. Fahey, Christopher E. Mason, Irene M. Min

**Affiliations:** Weill Cornell Medicine

## Abstract

ABSTRACT IMPACT: Here, we describe extensive sex-specific differences in the transcriptomes of pancreatic neuroendocrine tumors (PNETs). Given that the clinical course of PNETs differs by sex (female sex is associated with better survival), achieving a greater understanding of the specific molecular sexual dimorphisms is invaluable for advancing personalized treatment. OBJECTIVES/GOALS: Epidemiologic studies demonstrate that pancreatic neuroendocrine tumors (PNETs) exhibit sexual dimorphisms with regards to prognosis, disease recurrence, and complication rates. We sought to compare the transcription and DNA methylation landscapes of PNETs by sex, to elucidate molecular differences that may underlie this sex disparity. METHODS/STUDY POPULATION: RNAseq data was generated from PNETs surgically resected at our institution (9 Female; 12 Male patients). RNA was extracted with the RNeasy Mini Kit, stranded sequencing libraries were prepared with TruSeq, and paired end sequencing was done on the HiSeq 2500/4000 systems. Transcript-level quantification was performed with salmon, and DESeq2 was used for differential expression analysis. To account for significant variation due to covariates other than sex, surrogate variables were computed with the SVA package and adjusted for. The goseq package was used for gene set over representation analysis. Matched DNA methylation (DNAm) and RNAseq data was downloaded from GEO (16 F; 16 M). Raw DNAm data was processed with minfi. Differential methylation analysis was done with limma and bumphunter. Analysis was done in R. RESULTS/ANTICIPATED RESULTS: We found that 826 autosomal genes were differentially expressed (DE) by sex in PNETs (at FDR ≤0.1). Gene set over representation analysis performed on the DE genes revealed significant enrichment for several processes, including ‘ascorbate & aldarate metabolism’ and ‘positive regulation of ERK1 & ERK2 cascade’ (all FDR ≤0.1). When we compared DNAm profiles between sexes, we found 8 CpGs which were differentially methylated by sex (at FDR ≤0.1), 7 of which were proximal to genes. Methylation of one of the sex-associated CpGs, overlapping the gene TIMM8B, was found to be negatively correlated with gene expression (rho=-0.41; p-value=0.02). Interestingly, TIMM8B deletion has been previously reported in other non-pancreatic neuroendocrine tumors. There were no differentially methylated regions between sexes. DISCUSSION/SIGNIFICANCE OF FINDINGS: Our findings demonstrate that PNETs exhibit extensive sexual dimorphisms with regards to gene expression profiles but have largely congruent methylomes by sex. These molecular differences may contribute to the variability in clinical course between men and women, and their characterization is vital for the advancement of personalized medicine.

